# Abdominal Complications Related to Ventriculoperitoneal Shunt Placement: A Comprehensive Review of Literature

**DOI:** 10.7759/cureus.13230

**Published:** 2021-02-08

**Authors:** Leopoldo Mandic Ferreira Furtado, José Aloysio Da Costa Val Filho, Rodrigo Moreira Faleiro, José Antônio Lima Vieira, Aieska Kellen Dantas dos Santos

**Affiliations:** 1 Department of Pediatric Neurosurgery, Vila Da Serra Hospital, Nova Lima, BRA; 2 Department of Pediatric Neurosurgery, Vila da Serra Hospital, Nova Lima, BRA; 3 Department of Neurosurgery, João XXIII Hospital/Fundação Hospitalar do Estado de Minas Gerais (FHEMIG), Belo Horizonte, BRA

**Keywords:** catheters, hernia, hydrocephalus, risk factors, shunt infections, shunt migrations, abdominal pseudocyst, ascite

## Abstract

Ever since the shunt device became the gold standard treatment for hydrocephalus, complications due to infections and mechanical problems have increased while lives have been saved. In addition, abdominal complications have become an important issue as the peritoneum is now the main place to insert the distal catheter. The most common complications were abdominal pseudocyst, distal catheter migration, inguinal hernia, catheter disconnection, and intestinal obstruction. The pediatric population is more prone to develop most of these complications due to their rapidly growing body, weaker abdominal musculature, and increased intraabdominal pressure. The goal of this review was to study the main aspects associated with abdominal complications after ventriculoperitoneal shunt (VPS) insertion, including the pathophysiology, epidemiological aspects, as well as the rationale for management and prevention according to the current “state-of-the-art.” It is paramount to recognize the risk factors associated with various types of complications to manage them properly.

## Introduction and background

Hydrocephalus is the most common childhood brain disorder, affecting 1 per 500 births worldwide, and the incidence is highest in Latin America where it is estimated to occur in 360 per 100,000 births [1]. Historically, the diversion of cerebrospinal fluid (CSF) for the treatment of hydrocephalus was first performed at the end of the 19 century by Ferguson [2]. The use of the peritoneal cavity as the location for CSF absorption was introduced by Kaush in 1908, who used a rubber tube to divert the lateral ventricle to the peritoneum [3]. However, due to problems with production of this device, which presented numerous complications such as local obstruction, cracking, kinking, and a high rate of peritoneal inflammatory reaction, this procedure was abandoned in later decades [4]. With improvements of the device, the introduction of the first clinically successful unidirectional shunt was introduced by Nulsen and Spitz [5] in 1951. The first shunt made of silicone was developed by Pudenz [6] and Holter [3] in 1956. With the introduction of shunts made of silicone, less mechanical and inflammatory complications were observed as well as contributed to increasing the life span of shunts [7,8]. Additionally, the introduction of shunts impregnated with antibiotics has been shown to reduce the rate of shunt infection from 6.0% of standard VPS to 2.2% of antibiotic-impregnated VPS. Thus, shunts became the gold standard for the treatment of hydrocephalus, changing the natural history of hydrocephalus and significantly improving patient survival and decreasing the mortality rate [9].

**Figure 1:**
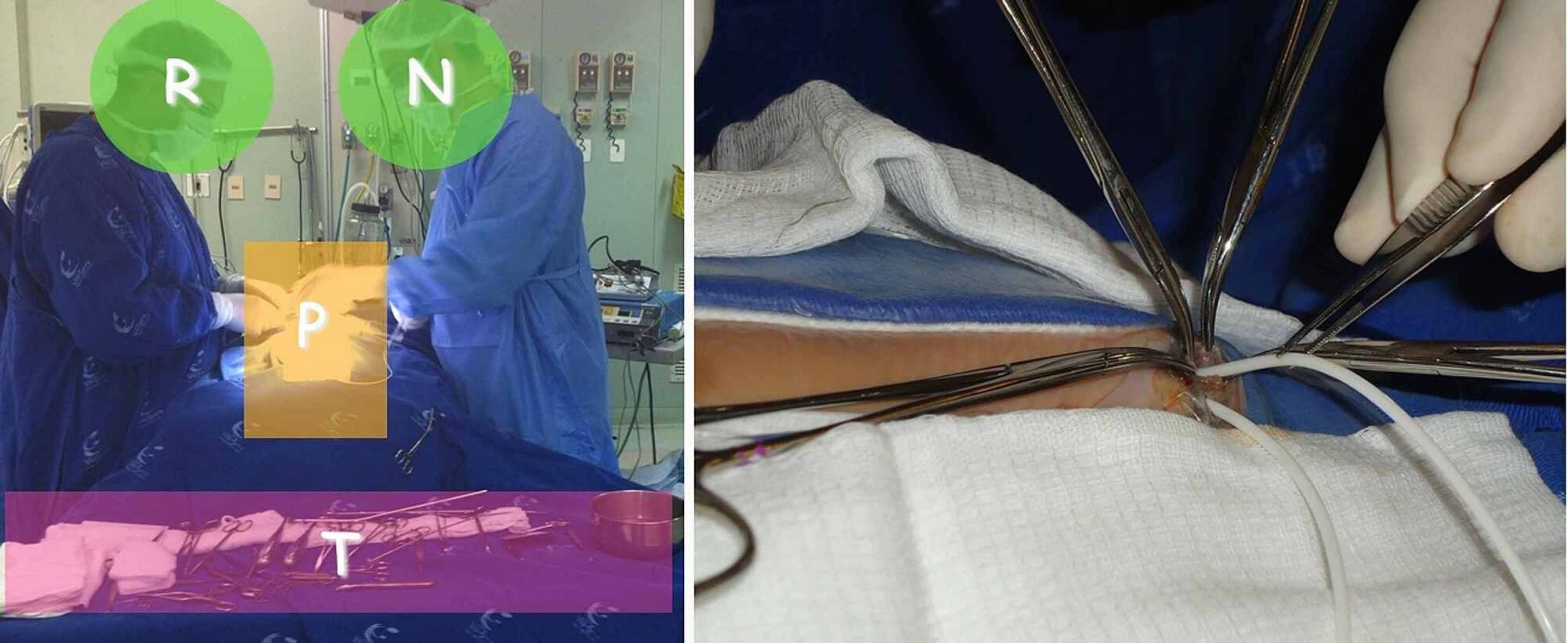
The ventriculoperitoneal shunt placement protocol. During surgical procedure, the neurosurgeon (N) and resident (R) are in front of each other, and the table with instruments (T) is positioned at the head of operating bed (P) (left). The distal catheter is introduced to peritoneum cavity avoiding touching the hardware and, instead, use delicate forceps (right).

Improvements in the shunt’s manufacturing have mainly impacted mechanical problems, whereas refinements in surgical technique have significantly reduced shunt complications. Through the remarkable work performed by Choux et al [10], a protocol for shunt implantation was developed. It addressed several issues such as reducing the number of individuals in the operating room, restricting manipulation of shunt hardware, and limiting the operation to only two most experienced neurosurgeons. After the protocol was implemented, a significant reduction in shunt infections was reported. This protocol was reproduced in our institution of pediatric neurosurgery and was also effective in reducing the occurrence of shunt infection from 5.7% to 1% (Figure 1) [11].

Nevertheless, the shunt itself carries a high rate of complication even with the best surgical technique, and it is expected that at least 60% of patients must undergo shunt revisions throughout their life. The most common causes of shunt failure are obstruction and mechanical failure, followed by excess drainage, loculations of the ventricular system, infections, and abdominal complications [12-15].

Although abdominal complications related to ventriculoperitoneal shunt (VPS) have been considered rare with heterogeneous presentation, incidence varied over the last decades, ranging from 5% to almost half of cases [16-22]. Primarily, abdominal complications related to VPS can be associated with infection or mechanical problems with the shunt. In some instances, technical inaccuracy also plays a role [23-25]. The most common types of abdominal complications are abdominal pseudocysts (APC), and shunt migrations, in which the distal shunt catheter could dislodge from the peritoneum cavity to other places and could perforating the abdominal wall or hollow viscus [26]. Some types of migrations, such as extrusion from the anus and perioral area, are exceedingly rare [20]. Intestinal obstruction by volvulus was reported, and the rate of inguinal hernia could increase due to increased abdominal pressure [17,25]. Complications related to fractures and disconnections accounted for 5%-16% of cases and are linked to deterioration of the biomaterials in combination with patient growth [7,14,27,28].

In this comprehensive review, we sought to describe the pathophysiology of the main types of abdominal complications as well as provide the current state-of-the-art treatment and the rationale for management protocols.

## Review

Pathophysiology and the role of the peritoneum cavity

The peritoneal cavity is the main place to divert the CSF due to this high ability to absorb fluids. Therefore, loss of absorption capacity of peritoneal membrane is the main cause of VPS failure related to APC [14,29].

Current knowledge about peritoneal dialysis failure due to peritoneal membrane compromise has been contributing to knowledge about abdominal complications related to VPS, because there is a close similarity between failure of peritoneum absorption during peritoneal dialysis and VPS failure due to the compromise of anatomical and physiological integrity of this membrane [14,30]. Primarily, this mesothelium is composed of a monolayer of mesothelial cells derived from mesoderm, which works as the first line of defense against microorganisms and chemical insults [30]. Furthermore, it synthesizes several growth factors and inflammatory mediators that play a role in repair and homeostasis, and the microvilli in its ultrastructure increase the absorption surface, with absorption through the lymphatics ranging from 0.5 ml/min to 1.5 ml/min [31]. Additionally, the peritoneum has a non-adhesive property due to the secretion of glycocalix, which decreases the damage from friction. It facilitates the movement of viscus and improves the longevity of a distal VPS catheter with minimal reaction [30].

Nevertheless, during an injury process with inflammation or infection, the mesothelium cells are prone to damage and suffer expoliation, exposing the underlying submesothelium, which triggers the inflammatory process. The replenishment of regular mesothelial cells could be compromised and affect catheter function due to the formation of fibrotic tissue [30]. Additionally, this decreases the ability to adequately absorb fluids and could sustain the inflammatory process due to constant inflammatory mediators being released by sub-mesothelium tissue. Therefore, this process contributes to persistent VPS failure [31].

An inflammatory reaction to a foreign body can be the etiology of VPS failure mainly due to fibrosis in the peritoneal space. The use of antifibrotic substances during shunt placement is a strategy to reduce this complication. Considering this process, an experimental study using rats was conducted by Aydoseli et al [32] to compare heparin, sodium hyaluronate/carboximethylcellulose, and icodextrin instillation in the peritoneal cavity during peritoneal catheter placement. They found that icodextrin, derived from glucose polymer, prevented the formation of intraperitoneal fibrosis and adhesions.

Diagnosis of abdominal complications

Clinical presentation: Given that abdominal complications of VPS are heterogeneous, many presentations can be seen, ranging from asymptomatic patients in whom fractured hardware may be incidentally diagnosed after an elective abdominal radiograph to an extruded catheter through abdominal skin that is clearly diagnosed during a physical exam [7,33].

Abdominal pseudocysts and ascites usually present with abdominal pain and distension due to increased abdominal volume [34]. Other reported presentations include shunt dysfunction symptoms such as fever and abdominal complaints of abdominal tenderness and acute abdomen [29,35-37]. Serious acute symptoms related to worsening hydrocephalus or central nervous system infection are also observed [38].

Imaging studies: Abdominal ultrasound can quantify the peritoneal volume and identify septations inside the cavity that may increase the chance of infection. This imaging can easily identify either pseudocysts or ascites related to VPS [36,39,40].

**Figure 2:**
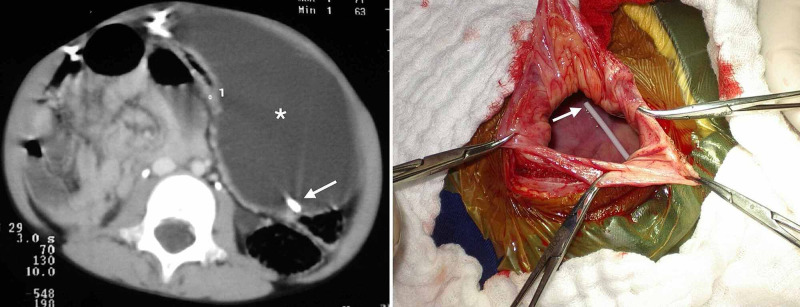
Abdominal pseudocyst. Computed tomography scan of abdomen shows a localized collection of fluid in left peritoneal cavity (*) (left). Intraoperative view of abdominal pseudocyst is shown with dilation of peritoneal cavity, and the distal catheter tip (white arrow) is seen (right).

Computerized tomography has been deemed the gold standard for the assessment of pseudocysts and ascites, providing a reliable view of the cavity as well as the position of the tip of the distal catheter (Figure 2) [38].

Laboratory tests: The main goal of laboratory testing is to determine whether there is an infection, mainly in cases of APC in which the sample is obtained of abdominal fluid as well as cerebrospinal fluid by shunt tap and culture of the tip of distal catheter [41]. Some studies have reported the importance of obtaining gram stain, culture, glucose, protein, and cell counts, including differential of both fluid samples [41].

Main types of abdominal complications

Abdominal Pseudocyst

The first description of APC following VPS was by Harsh in 1954 [42]. Although this complication is infrequent and has been reported with an incidence from 0.25% to 10% in the literature, it is considered important and not completely understood [34,37,38,43]. There are several hypotheses to explain its origin such as indolent low-level shunt infection, mainly due to Staphylococcus epidermidis or Propionibacterium acnes, degradation of distal catheter, or in rare cases, allergy to polydimethylsiloxane, called silicone elastomer, which is present in catheters [6,34,44]. The term pseudocyst refers to the absence of mesothelium and inflammatory reaction of the wall. Biopsy of peritoneal tissue in the vicinity of the catheter may reveal a chronic inflammatory reaction such as a foreign body reaction [34]. The interval between shunt placement and APC is reported to be 16 months in the majority of cases [45].

Gmeiner et al [38] performed a retrospective study considering at least 20 years of follow-up of patients who underwent distal catheter revisions and analyzed a cohort of 138 patients. They found APC in 12.5% of the patients, and there was no statistical correlation with etiology or the time of shunt implantation. Furthermore, indolent low-level shunt infection was regarded as the main explanation for the high rate of APC in this study, as more frequent shunt infection was observed. They concluded that APC was the main complication observed over the long term in patients with shunts.

The association of APC with shunt infection was corroborated by Salomao et al [37], who conducted a retrospective study analyzing 18 patients with APC. Their study suggested that examination of the shunt tip was reliable and more representative than CSF to identify infection. They stated that the best approach was to convert the shunt to external ventricle drainage (EVD), and shunt infection was identified in 66% of catheter tip cultures.

**Figure 3:**
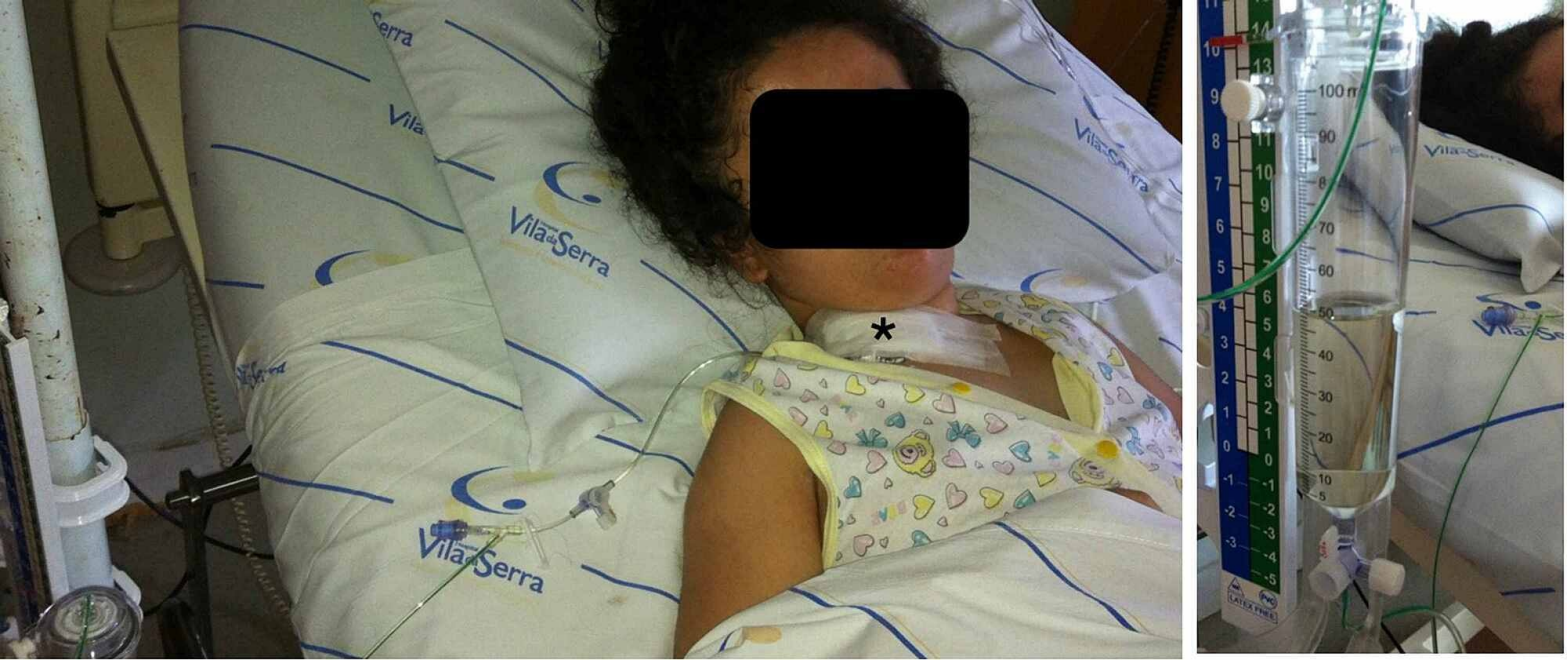
Conversion of VPS into EVD. Patient with abdominal pseudocyst underwent an externalization of distal VPS catheter at the level of the clavicular region (*) (left). CSF depicting clear aspect (right). VPS: ventriculoperitoneal shunt; EVD: external ventricle drainage; CSF: cerebrospinal fluid.

The majority of algorithms recommend evaluation of the shunt infection before externalizing the VPS to EVD [37,41,45]. One option is to puncture the APC and obtain a fluid sample for analysis [37]. If no infection is found, a laparoscopic approach could be indicated to reposition the catheter inside the peritoneum. Another option is to simply puncture the APC to relieve pressure [46]. This approach often leads to recurrence of APC [41]. Laparoscopic management of APC is described as a valid approach to relieve intrabdominal pressure and reposition the tip of distal catheter to better absorb CSF [47]. Given that APC is associated with low-level infection in most cases, we externalize the shunt to EVD at the level of clavicle in our practice (Figure 3).

During the externalization of VPS, the distal catheter is removed from the peritoneal cavity and connected to an external system to collect CSF. CSF can then be easily collected for laboratory analysis. The rationale for this approach is to continue treating the hydrocephalus and stop the infection process at the same time. Furthermore, abdominal pressure improves, and the cause of infection can be diagnosed. After treatment with antibiotics, a new surgery is needed to convert the EVD back to an internal shunt. Several studies report similar findings using this technique, owing to the advantages [35-37,40].

After the resolution of APC and treatment of infection, the new VPS could be placed on the opposite side [37]. Locations such as atrium [38], pleura [2], and gallbladder have been frequently described [2]. The retro-hepatic region was described as an alternative approach by Oliveira et al [41]. Another alternative could be another ventriculostomy (ETV) in selected cases, ruling out the use of a shunt altogether [41].

In our experience, after the first shunt failure and in patients with EVD, we speak to the parents of the patient about the advantages of ETV. Magnetic resonance imaging is effective in assessing ventricle dilation and anatomy of the third ventricle. If we can predict at least 50% chance of success, we perform ETV [48].

Ascites

**Figure 4:**
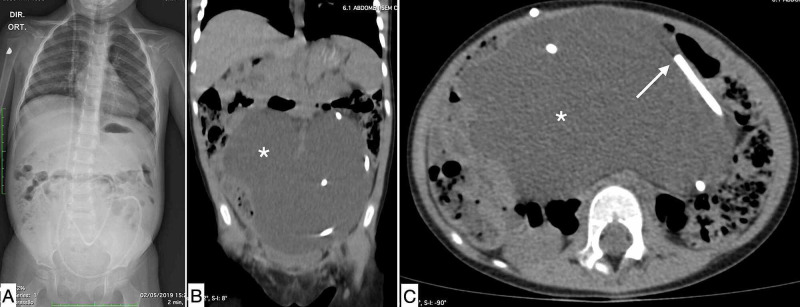
Ascite as an abdominal complication related to VPS. (A) Abdominal X-ray shows its increased size and the distal VPS catheter; (B) coronal view of computed tomography scan of the abdomen shows a homogeneous distribution of peritoneal fluid (*); (C) axial view of computed tomography scan of abdomen depicted the tip of distal catheter (white arrow). VPS: ventriculoperitoneal shunt.

Ascites are less frequent than APC, and the main etiology is related to an increased level of protein in the CSF that interferes with peritoneal absorption [15]. During imaging, homogeneous dilation of the peritoneal cavity with no loculations is a feature of ascites (Figure 4).

Kariyatil et al [46] reported a retrospective analysis of 30 years of experience, and several differences between APC and ascites related to VPS were reported. Patients with ascites presented with abdominal distension and discomfort. Otherwise, 40% of patients with APC have symptoms of shunt obstruction. Furthermore, analyses of fluid from ascites and APC can differentiate the etiology. Ascites displayed a transudate pattern of fluid with < 3 g/dl of protein content of abdominal fluid, in contrast to APC that were positive for infection. Therefore, the authors pointed out that ascites and APC were pathogenetically different. APC was related to an inflammation reaction and loculation of peritoneal cavity, and ascites originated from excess CSF production exceeding the peritoneal absorption.

Despite reports that recommended placing the shunt in another site (such as atrium, pleura, or gallbladder) whether ascites is suspected [23,43], our experience shows good outcomes with externalization of distal catheter to an EVD and reimplantation in the peritoneum cavity or consideration of ETV.

Migration of distal catheter

**Figure 5:**
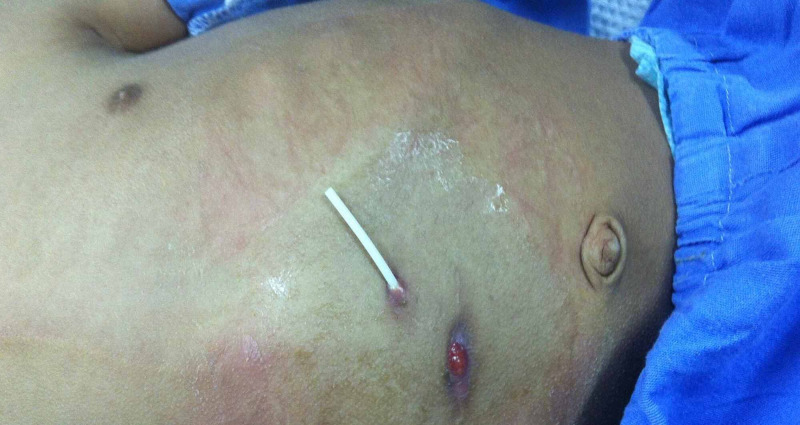
Migration of distal VPS catheter. The image shows a patient with abdominal wall perforation by a distal ventriculoperitoneal catheter. An anomalous scar formation was observed in the surgical incision. VPS: ventriculoperitoneal shunt.

Cases of migration of distal catheters have been reported with great variability, from perforation of hollow viscus to exteriorization to a natural body opening such as the mouth, urethra, and anus; or to perforation of the abdominal wall (Figure 5) [20,22,25].

A worst-case scenario would be perforation of the colon, which could lead to a retrograde translocation of bacteria (mainly Escherichia coli) that could ascend through the device, causing ventriculitis, meningitis, and sepsis. This complication had a high mortality rate of 15% [22]. Despite the rare incidence of bowel perforation by peritoneal catheters, with one study reporting it in 0.07% of cases, the mortality rate was 15% [25].

Although the exact mechanism is not fully understood, the main hypothesis is that there is a formation of fibrous area between the tip of the catheter and abdominal tissue. In addition to the continuous pressure of CSF, this fibrous tissue could soften the viscus in the body or abdominal wall, leading to perforation and further migration of the catheter [33].

Allouh et al [33] contributed a meaningful review of all case reports of distal catheter migration during a five-year period and created a classification of three anatomical patterns of migration. They analyzed 323 indexed articles in PubMed with 437 cases of distal catheter migration and divided cases into three types of migration. Type I or internal migration was when the catheter perforated the viscus with no exteriorization. The authors found this complication in 164 patients and reported that this complication was most common in adults. Type II or external migration was when the catheter penetrated the body wall either completely or subcutaneously. This type was recorded in 144 cases. Finally, in Type III or compound migration, the catheter perforated the hollow viscus and reached a natural body opening. It could exteriorize at the anus, mouth, and less frequently, urethra and vagina. The last two types of migration were observed mainly in children, most likely owing to the fragility of the muscles of the abdomen wall. The rationale of management of catheter migration is to address the compromised catheter and resolve the viscus perforation with the aid of a pediatric surgeon.

Hernia and hydrocele

The incidence of bilateral inguinal hernia was 20%. In unilateral cases, herniation in the contralateral and asymptomatic side was often found on examination [25]. The most accepted theory of physiopathology is related to increased intraabdominal pressure due to CSF in the peritoneal cavity [25]. Incisional hernia was also reported [16].

Burhan et al [25] described their experience of 16 cases with abdominal complications after VPS. They found a mean interval of 38 days between shunt implantation and the appearance of hernia or hydrocele. Overall, they also reported that abdominal complications generally appeared within eight months.

**Figure 6:**
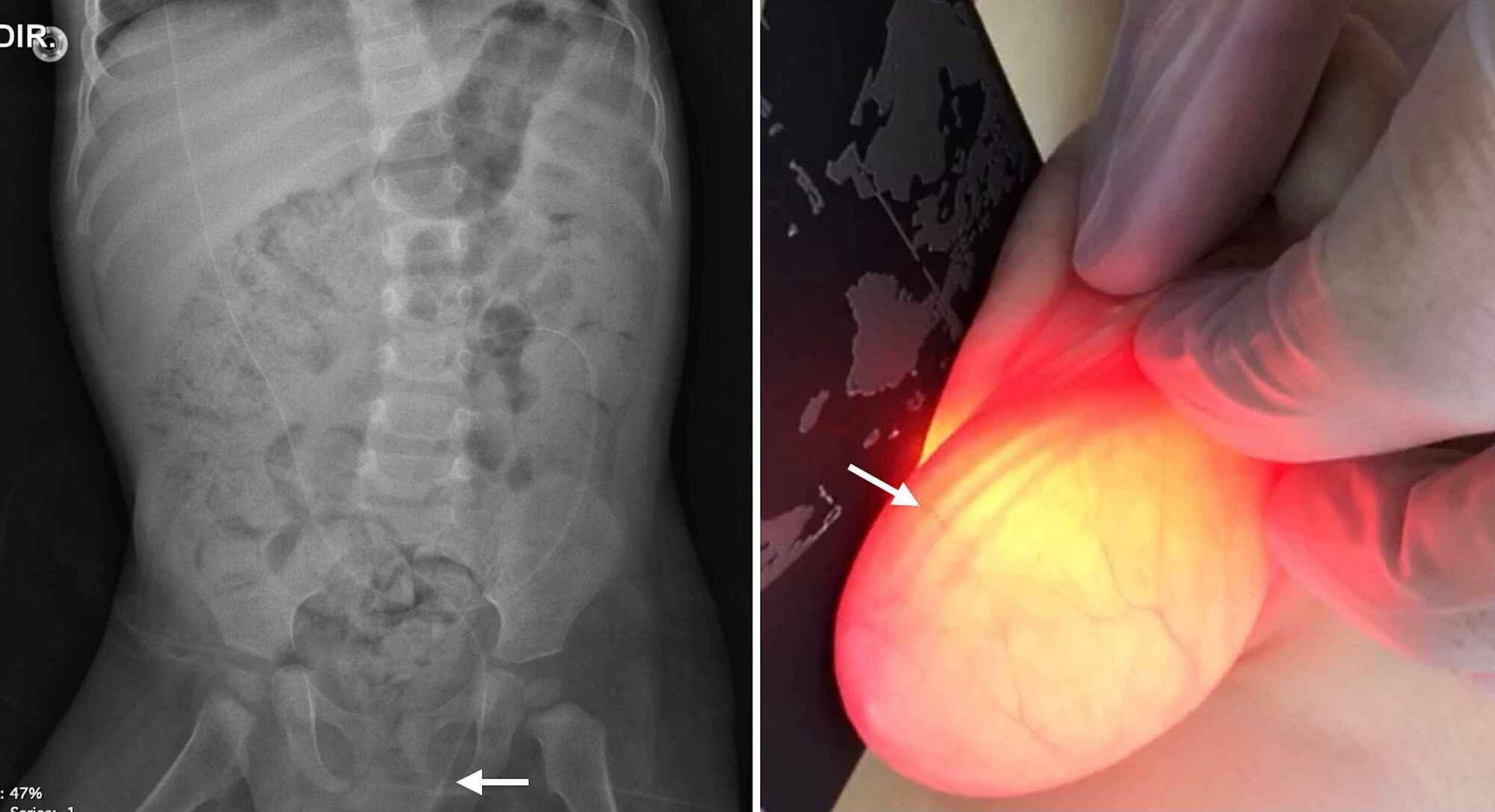
Migration of distal VPS catheter to scrotum. Abdominal X-ray shows the migration of distal catheter of VPS (white arrow) to the left scrotum (left). During the physical exam, a transillumination of the left scrotum allows to perceive the entanglement of distal catheter inside of this region (right). VPS: ventriculoperitoneal shunt.

The approach to resolve this complication includes hernia treatment and repositioning of distal catheter in the peritoneal cavity. In our experience, if ascites are also present, we exteriorize the distal catheter to an EVD and perform an ETV. In case of failure, a new VPS is placed in an opposite location (Figure 6).

*Intestinal obstruction* 

This complication is considered exceedingly rare and with paucity reports in the literature, and the most common causes are volvulus followed by twisting of distal catheter around the hollow bowel and peritoneum adhesions [17,37]. A greater omentum was often implicated in the obstruction, according to observation of experienced pediatric neurosurgeons [49]. Intestinal obstruction and shunt malfunction due to an entanglement should be carefully managed, and laparoscopy could play an important role in diagnosis and treatment [17]. Bacterial translocation into the CNS was reported and is a cause of more severe cases. According to the algorithm proposed by Zhao et al [17], the surgical management of intestinal obstruction after VPS should consider whether there is complete intestinal obstruction. If CSF infection is diagnosed, they recommended removal of the entire device or proceeding with distal catheter externalization. They also considered ETV in cases of obstructive hydrocephalus or replacement by a new VPS or a ventriculo-atrial shunt.

Fracture and/or disconnection

**Figure 7:**
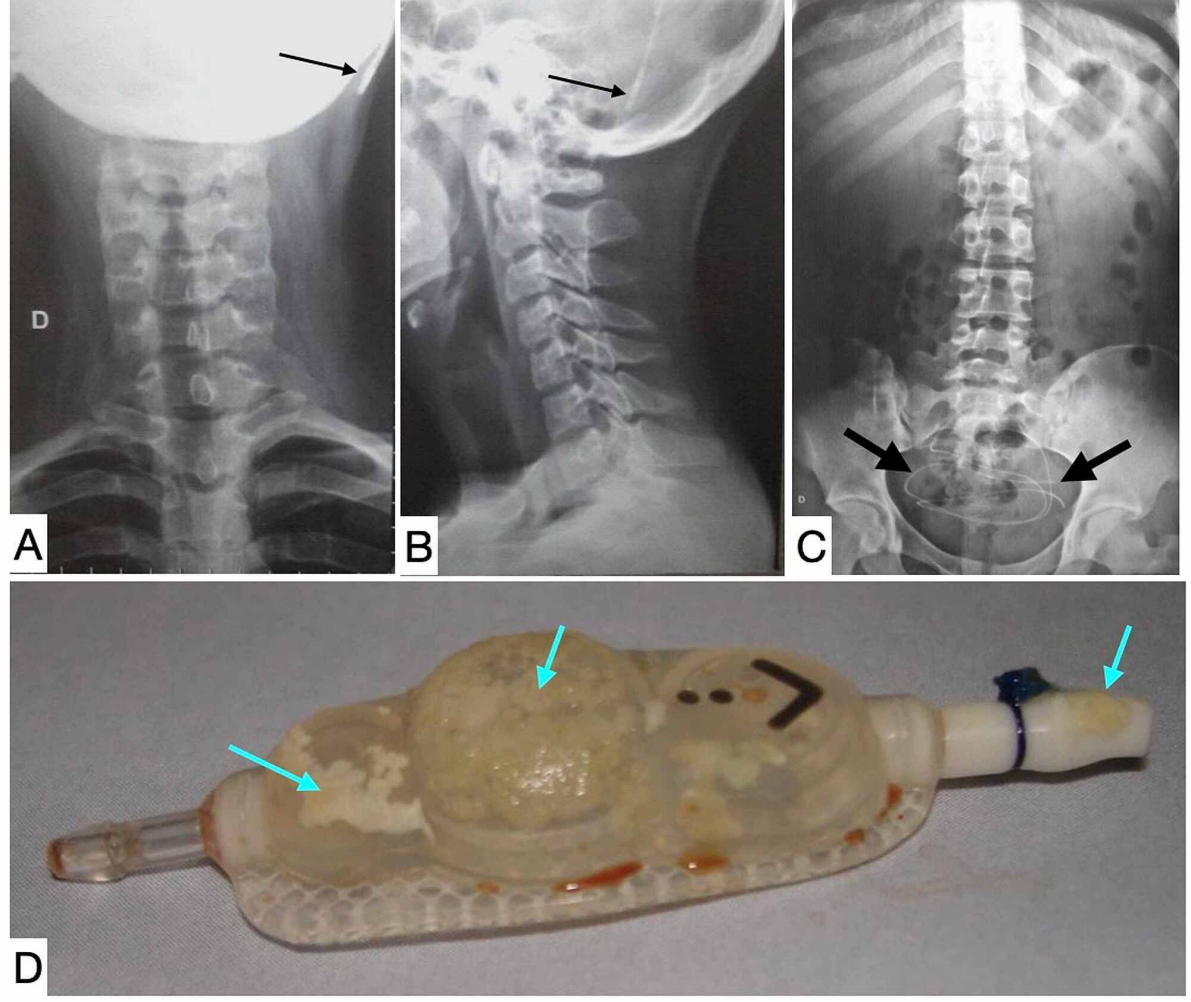
Shunt disconnection. The cervical X-ray displayed a shunt disconnection at the level of left retromastoid region (thin black arrow) in both projections anteroposterior (A) and lateral(B). The abdominal X-ray shows a complete migration of the distal catheter (thick black arrow) (C). The shunt mineralization as well of distal catheter was shown in this other patient (blue arrows) (D).

The main cause of fracture and disconnection is the growth of pediatric patients leading to a stretching of the catheter, mainly in the connections on the device. Formation of scar tissue at the connection points can be seen, anchoring the device in place. Another cause of fracture or disconnection is mineralization and deterioration of the silicone in the device, decreasing elasticity and contributing to the fracture of a catheter (Figure 7) [27,38].

In some cases, disconnection and withdrawal of the device from the peritoneum are incidentally discovered with no symptoms. According to Clyde and Albright [7], this could be explained by an underlying arrested hydrocephalus or persistence of a fibrous tract that maintained the CSF diversion to the peritoneum. They recommended a shuntogram as a tool to differentiate functional and nonfunctional fibrous tracts.

## Conclusions

Hydrocephalus is an especially important neurological disorder, and VPS remains the standard of care in its treatment even after the acceptance of ETV in recent decades. Despite abdominal complications related to VPS being considered rare in some reports, this present review on this topic demonstrates that these complications are most likely underreported in many neurosurgery institutions. Close surveillance of all patients after undergoing shunt placement is paramount for prompt diagnosis and best management. Further multicentric studies regarding the predicting factors associated with abdominal complications related to VPS will be needed in order to achieve more evidence on this topic.
